# Post-traumatic growth after cancer: a scoping review of qualitative research

**DOI:** 10.1007/s00520-021-06253-2

**Published:** 2021-05-20

**Authors:** Fiona Menger, Nurul Asyiqin Mohammed Halim, Ben Rimmer, Linda Sharp

**Affiliations:** 1 School of Education, Communication and Language Sciences, Newcastle University, Newcastle upon Tyne, UK; 2 Newcastle University Medicine Malaysia, Johor, Malaysia; 3 Population Health Sciences Institute, Newcastle University Centre for Cancer, Newcastle University, Newcastle upon Tyne, UK

**Keywords:** Post-traumatic growth, Cancer, Survivorship, Coping

## Abstract

**Purpose:**

Interest is growing in post-traumatic growth (PTG) after cancer prompted, in part, by observations of positive associations with health-related quality of life. Qualitative research provides valuable insight into survivors’ experiences. We conducted a scoping review of qualitative evidence on PTG in cancer, determining the number, nature, range and scope of studies, and gaps in the literature.

**Methods:**

We systematically searched Medline, Scopus, CINAHL, Web of Science, and PsycINFO for qualitative research exploring positive changes after cancer published from 1996. From eligible studies, we extracted: terms used for PTG; design, methodological orientation, and techniques, and participant characteristics. Using descriptive mapping, we explored whether study findings fit within Tedeschi and Calhoun’s PTG framework, and evidence for unique positive changes post-cancer.

**Results:**

Twenty-eight studies were eligible. Cancer sites included were: breast, 14; mixed, 6; haematological, 4; head and neck cancer, 2; bone, 1, and testis, 1. Multiple studies were conducted in: the USA (12), Australia (3), Iran (2), and the UK (2). Twenty-three studies collected data using individual interviews (21) or focus groups (2). Definitions of PTG varied. Studies largely focused on descriptive accounts of PTG. Findings mapped onto existing PTG dimensions; health behaviour changes were often reported, under ‘new possibilities’.

**Conclusions:**

A range of PTG outcomes can occur after cancer. Positive health behaviour changes warrant further exploration. Future research should include more diverse patient populations, collect longitudinal data, and focus on pathways towards positive changes.

**Supplementary Information:**

The online version contains supplementary material available at 10.1007/s00520-021-06253-2.

## Introduction

The increasing number of cancer survivors worldwide [1, 2] has led to greater focus over the past 10–20 years on investigating and understanding survivors’ needs, experiences, and outcomes. Most of that research has focused on identifying problems, limitations, and adverse impacts of cancer on people’s lives [3], with the goal of developing services, supports, and interventions to ameliorate these impacts. However, interest has also grown in exploring the potential for survivors to experience positive consequences of their illness. For example, survivors can report positive behavioural, emotional, or cognitive changes following cancer, such as an enhanced sense of appreciation of life, more meaningful relationships, or a richer existential and spiritual life [4–6].

These changes are commonly referred to as post-traumatic growth (PTG). The term was first described by Tedeschi and Calhoun in relation to responses to wider experiences of trauma, e.g., survivorship of a natural disaster [7]. These authors argue that traumatic events (such as cancer and its treatment) are, in themselves, insufficient to cause PTG. Instead, an individual must reflect on their experiences and seek to find meaning in them, i.e., growth arises from adaptation to the trauma and rebuilding one’s sense of the world [8].

Quantitative data suggests that a substantial proportion of cancer survivors experience PTG [9–11]. Moderate-high growth has been reported in around 60% of survivors [12, 13]. In terms of correlates, age, sex, time since diagnosis, type of treatment, and cancer stage have been associated with PTG, but evidence is inconsistent [9, 10]. More consistent positive relationships with social support and resilience have been reported [9, 14] and PTG is positively associated with health-related quality of life [15]. However, while this body of research has been valuable in quantifying the extent of PTG among survivors, it has limitations, including cross-sectional designs and significant heterogeneity [15, 16]. Nor can it shed light on pathways by which PTG develops or whether any aspects of the experience of PTG are specific to cancer.

Qualitative research methods seek to understand people’s experiences, providing detailed, rich, and deep data. By exploring how a phenomenon manifests and is experienced and by whom, the context in which it occurs, and what may influence or affect that experience, qualitative data — and reviews of the body of qualitative data — can help identify gaps in knowledge and understanding [17]. Further, it can form an essential underpinning in the rigorous development of interventions [18, 19].

We undertook a scoping review to address the following research questions:

1. What is the extent, range, and nature of existing qualitative research on PTG in survivors of cancer?
2. Do findings from existing qualitative research on PTG after cancer fit with wider descriptions of PTG or are there unique positive changes after cancer?

## Methods

Where there is lack of clarity on the extent of literature on a topic or on the types of evidence available, a scoping review is appropriate [20]. To address our first research question, we were guided by Arksey and O’Malley’s stages of a scoping review: identifying relevant studies, study selection, charting the data, and collating, summarising, and reporting the results [21]. (We had already defined our research questions, so the first stage of Arseky & O’Malley was not relevant.) To address our second research question, which leaned more towards qualitative evidence synthesis, we followed the initial stages of a pragmatic descriptive mapping approach [22]. For review conduct and reporting, we were guided by Preferred Reporting Items for Systematic Reviews and Meta-Analyses extension for Scoping Reviews (PRISMA-ScR) [23].

### Identifying relevant studies

We searched Medline, Scopus, CINAHL, Web of Science, and PsycINFO from 1996 onwards, as this year marked the first paper published by Tedeschi and Calhoun using the term post-traumatic growth [24]. A combination of MeSH headings and text words were used, representing the phenomenon of interest (PTG), the trauma of interest (cancer), and the study methodology (qualitative). The search was executed on 5 July 2019, with database alerts used to identify any further studies by 10 April 2020. Online Resource 1 provides a table illustrating an example search strategy.

### Study selection

After initially scanning the search results, we defined our inclusion criteria as follows:

- Primary qualitative research based on original data, or mixed methods studies which reported qualitative data separately, reported in full and published in English;
- Studies which aimed to identify or explain PTG; recognising not all have used the same terminology, we also included those that did not use this term but used terms that could be used synonymously (e.g., stress-related growth and adversarial growth);
- Studies including participants with any type of cancer diagnosed in adulthood. Survivors of childhood cancer are likely to experience unique issues and challenges due to their life stage and thus their experiences of PTG may be different;
- Studies conducted with individuals who had sufficient time following treatment for rumination and reflection to occur and for PTG to develop. Guided by Tedeschi and Calhoun’s definition of PTG [25], participants had to be at least 6 months post-diagnosis of cancer or 3 months after completion of treatment;
- Studies which recruited cancer survivors and others, but where data could be separately extracted from survivor participants.

Unpublished doctoral dissertations were included, unless a peer reviewed paper reporting the results had also been identified. We excluded studies which comprised participants described as being at the end of life or undergoing palliative care, as their experiences of PTG may be quite specific. We also excluded studies which focused on general experiences of cancer survivorship (rather than PTG) and those which examined PTG solely from the perspective of those other than the cancer survivor (e.g., spouses and informal carers). Finally, we excluded studies which set out to consider only one aspect of positive change.

Following deduplication of search results, titles and abstracts were screened in Rayyan [26], independently by two authors (FM, NMH). Any conflicts were discussed. Full text review of potentially eligible studies was conducted by the same two authors. Any conflicts were resolved by discussion or consultation with a third author (LS). Reference lists of eligible studies were hand checked for any additional studies which had been missed.

### Data charting, summary, and synthesis

To address our first research question, we charted data on study aims and characteristics (including where conducted, cancer sites, and participants’ characteristics). Terms used to describe PTG or the construct considered in the paper, and any definitions provided, were documented. This was initially conducted by one author (NMH) and then checked by a second author (BR). Following initial charting, we tabulated characteristics of all included studies to summarise the extent, range, and nature of existing qualitative research on PTG after cancer.

We then examined each charted definition to determine whether the authors’ discussion of the construct being examined contained reference to positive changes: (1) following a life struggle or traumatic event; (2) being unplanned or unexpected; (3) occurring a period of time after the trauma or following a period of rumination or reflection, and (4) being a process as well as outcome [25].

We charted information on methodological stance and data collection and analysis methods using Bradbury et al.’s Qualitative Research Level of Alignment Wheel (QR_LAW™) [27]. This enabled comparison of the philosophical orientation of the work and the qualitative methods/techniques used to investigate PTG. We also collated (where reported) example questions that reflected how data was elicited from study participants.

To address our second research question, findings from eligible studies (textual descriptions from the results section or elsewhere in the paper and quotes) were collated and mapped to Tedeschi and Calhoun’s PTG outcomes: relating to others, new possibilities, personal strength, spiritual change, and appreciation of life. Any findings which did not fit within one of these categories were collated separately. This was done by one author (NMH) and checked by two others (FM, BR) with discussion, as required, to reach consensus. For mixed methods studies, only qualitative findings were abstracted.

## Results

### Search results

**Fig. 1:**
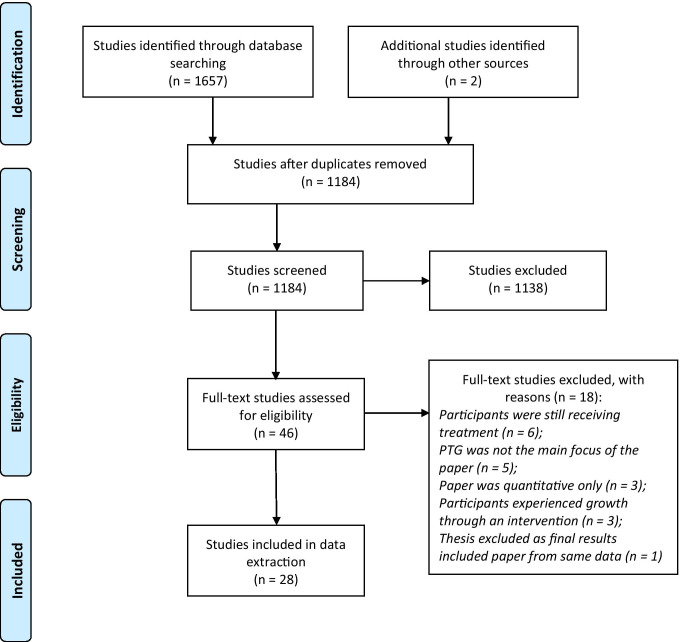
PRISMA flow diagram of eligible studies

The searches resulted in 1657 citations with an additional two studies identified through database alerts. Following deduplication, titles and abstracts of 1184 records were screened. Full texts of 46 studies were reviewed and, of these, 28 studies were eligible for inclusion (Fig. 1).

### Study aims

Of the 28 studies [28–55], five aimed to explore both positive and negative experiences [28, 32, 41, 49, 54] and 15 were focused only on positive experiences, either framed in terms of PTG or positive constructs such as meaning-making [30, 33–38, 43, 45–48, 51, 53, 55]. In six studies, the stated aim was broader (e.g., the original research question had a focus on areas such as adjustment or lasting life changes after cancer) but results were reported with a focus on the positive changes found [29, 31, 39, 42, 44, 52]. Two studies had very specific aims — exploring differences between people with cancer and their spouses in terms of growth [40] and an examination of growth in relation to other constructs, such as lifestyle changes [50].

### Study characteristics

Table 1 summarises characteristics across all studies. Tables 2 and 3 show, for each study, characteristics of participants and aims, orientations, and techniques, respectively.

**Table 1:** Summary of included study characteristics (*n* = 28)

Study characteristics	*n* (%)	Study characteristics	*n* (%)
Country		Sample size	
USA	12 (42.9)	≤ 15	11 (39.2)
Australia	3 (10.7)	16–50	9 (32.2)
Iran	2 (7.1)	> 51	8 (28.6)
UK	2 (7.1)	Study design	
Other^1^ (1 paper each)	9 (32.2)	Qualitative	16 (57.1)
Cancer site		Mixed methods	12 (42.9)
Breast	14 (50)	Orientation (methodological or philosophical position)	
Mixed	6 (21.4)	Generic qualitative	13 (46.4)
Haematological	4 (14.3)	Phenomenology	9 (32.2)
Head and neck	2 (7.1)	Grounded theory	3 (10.7)
Bone	1 (3.6)	Narrative	2 (7.1)
Testicular	1 (3.6)	Case study	1 (3.6)
Findings in relation to PTG outcomes^a^		Data generation technique^b,c^	
Relating to others	26 (92.9)	Individual interviews	21 (72.4)
New opportunities	23 (82.1)	Other techniques	5 (17.2)
Personal strength	27 (96.4)	Focus groups	2 (6.9)
Spiritual change	19 (67.9)	Narrative interview	1 (3.5)
Appreciation of life	25 (89.3)	Data analysis technique^c^	
Number of PTG outcomes reported		Thematic analysis	9 (32.2)
Five	16 (57.1)	Content analysis	5 (17.9)
Four	6 (21.4)	Phenomenological analysis	4 (14.3)
Three	4 (14.3)	Constant comparison	4 (14.3)
Two	2 (7.1)	Other	6 (21.3)

^a^Other countries: China, France, India, Italy, Japan, North America, Norway, Switzerland, and Turkey

^b^One study conducted both interviews and focus groups [46] and has been counted twice

^c^Data generation and analysis techniques were mapped to the QR_LAW[25]

**Table 2:** Characteristics of study participants

Source (country)	Sample size	Cancer site	Age^a^	Sex	Time since diagnosis^b^	Time since treatment^b^
Fromm et al. [28] (USA)	90	Haematological	38.8	M (58%), F (42%)	28.9 months	49.5 months
Carpenter et al. [29] (USA)	60	Breast	53.7	F (100%)	30.8 months	27.1 months
McGrath [38] (Australia)	12	Haematological	25–60	M (66.7%), F (33.3%)	3–15 years	1–10 years
Dahan et al. [39] (USA)	6	Haematological	50–66	M (50%), F (50%)	NR	> 3 months
Ruf et al. [40] (Switzerland)	31	Head and neck	58.2	M (100%)	3.7 years	> 6 months
Hegelson [41] (USA)	180	Breast	59.43	F (100%)	10.58 years	NR
Sadler-Gerhardt et al. [42] (USA)	8	Breast	30–80	F (100%)	NR	10 months–5 years
Thambyrajah et al. [43] (UK)	20	Head and neck	67	M (50%), F (50%)	NR	6–14 months
Bishop et al. [44] (North America)	30	Haematological	51.3	M (46.7%), F (53.3%)	NR	12.9 years
Morris et al. [45] (Australia)	209	Mixed	62.99	NR	2.9 years	NR
Hoggan [46] (USA)	18	Breast	37–65	F (100%)	NR	3–7 years
Lelorain et al. [47] (France)	28	Breast	NR	F (100%)	5–15 years	NR
Tsuchiya et al. [30] (Japan)	10	Breast	53.3	F (100%)	NR	5.2 years
Documet et al. [48] (USA)	112	Breast	34–81	F (100%)	1.53–29.36 years	NR
Frye [49] (USA)	6	Mixed	60–86	M (16.7%), F (83.3%)	14–32 years	NR
Triplett [50] (USA)	87	Breast	18–45	F (100%)	12 months	NR
Connerty et al. [51] (Australia)	15	Mixed	36–85	M (53.3%), F (46.7%)	15 years	NR
Fauske et al. [52] (Norway)	8	Bone	18–50	M (50%), F (50%)	3–10 years	NR
Mehrabi et al. [53] (Iran)	18	Breast	31–65	F (100%)	NR	3–6 months
Cheng et al. [54] (China)	29^c^	Breast	53.9	F (100%)	6–180 months	NR
Martino et al. [55] (Italy)	12	Mixed	25–70	M (25%), F (75%)	NR	3 years
Matheson et al. [31] (UK)	18	Testicular	34	M (100%)	NR	6 months
Hoogland [32] (USA)	56	Mixed	72.45	M (39.3%), F (60.7%)	36.71 months	NR
Barthakur et al. [33] (India)	15	Breast	57	F (100%)	9.3 years	> 6 months
Raque-Bogdan et al. [34] (USA)	13	Breast	34	F (100%)	3.5 years	NR
Adorno et al. [35] (USA)	5149	Mixed	NR	M (36%), F (64%)	2–10 years	NR
Inan et al. [36] (Turkey)	13	Breast	48.7	F (100%)	NR	7–22 months
Fallah et al. [37] (Iran)	23	Breast	46.22	F (100%)	26.96 months	NR

^a^Age is reported as the mean. Where the mean is not available, the range is given

^b^Time since diagnosis and treatment is reported as the mean. Where the mean is not available, the range is given, or time is denoted from the inclusion criteria

^c^A sub-sample of 29 participants were involved in the qualitative stage (full sample *n* = 100)

^d^*NR*, not reported

**Table 3:** Study aims, orientations, and techniques

Source (country)	Study aim	Study design	Orientation	Data collection method (Qual)	Example question framing	Data analysis method
Fromm et al. [28] (USA)	To explore the types of positive and negative consequences experienced following bone marrow transplantation	Mixed methods	Generic qualitative	Semi-structured interview	Have you noticed any changes in your relationships with other people since your transplant?	Thematic analysis
Carpenter et al. [29] (USA)	To examine differences in self-transformation and factors associated with self-transformation in breast cancer survivors	Mixed methods	Narrative	Semi-structured interview	How have you or your feelings about yourself changed since your breast cancer diagnosis?	Narrative analysis
McGrath [38] (Australia)	To explore positive outcomes within the notion of spirituality in survivors of haematological malignancies	Qualitative	Phenomenology	Open-ended interview	Could you tell me of your experience, in your own words and in your own way, from the time you became aware that you were ill? In particular, I am interested to hear about how that has changed how you see the world and what you believe is important?	Thematic analysis
Dahan et al. [39] (USA)	To understand the emotional impact of multiple myeloma, as well as the impact of its principle treatment, peripheral blood stem cell transplant (PBSCT)	Qualitative	Grounded theory	Semi-structured interview	Not reported	Constant comparison
Ruf et al. [40] (Switzerland)	To explore whether the frequency of positive changes and the areas in which positive changes are found differ between patients and their women partners	Qualitative	Generic qualitative	Structured psychiatric interview	Did you experience positive changes in your life since your cancer diagnosis?	Content analysis
Hegelson [41] (USA)	To learn how women viewed the experience 10 years later in terms of both benefits and costs following breast cancer	Mixed methods	Generic qualitative	Open-ended interview	Now that it is more than 10 years later, do you see that breast cancer has had any lasting effects on your life?	Coding framework
Sadler-Gerhardt et al. [42] (USA)	To investigate the lived experience of women surviving breast cancer by inviting them to tell about if and how they had changed and what meaning they were making of the process	Qualitative	Phenomenology	Semi-structured interview	Not reported	Thematic analysis
Thambyrajah et al. [43] (UK)	To explore whether patients with head and neck cancer experience positive consequences posttreatment and to investigate the nature of any benefit finding	Qualitative	Generic qualitative	Semi-structured interview	What good things, if any, do you think have come out of your illness?	Framework analysis
Bishop et al. [44] (North America)	To gain a deeper understanding of lasting life changes experienced by cancer survivors following blood and marrow transplantation	Qualitative	Generic qualitative	Semi-structured interview	What would you describe as the most significant long-lasting positive change in your life since your cancer experience?	Content analysis
Morris et al. [45] (Australia)	To ascertain the salience and prevalence of different PTG domains with cancer survivors	Mixed methods	Phenomenology	Open-ended survey question	Please feel free to add any comments that relate to the way in which you are dealing with (or have dealt with) being diagnosed with cancer or to the changes in your life that may have happened since your diagnosis	Thematic analysis
Hoggan [46] (USA)	Based on the assumption that challenging life events hold the potential for personal growth, this sought to better understand this process of growth as a learning experience	Mixed methods	Case study	Semi-structured interview, focus group	In what ways did you change because of your breast cancer experience?	Constant comparison
Lelorain et al. [47] (France)	To explore the emergence of PTG in breast cancer survivor narratives concerning the changes caused by the cancer in their lives	Mixed methods	Generic qualitative	Open-ended interview	Do you have the feeling that this cancer has changed something in your life or in yourself or, on the contrary, do you have the feeling that in the end nothing has really changed in your life or in yourself because of cancer?	Discourse/text analysis
Tsuchiya et al. [30] (Japan)	To describe positive changes following breast cancer diagnosis, together with factors affecting the changes, among Japanese breast cancer survivors	Qualitative	Generic qualitative	Semi-structured interview	Not reported	Thematic analysis
Documet et al. [48] (USA)	To explore (a) how women who were diagnosed with breast cancer (BC) defined themselves as survivors and when this occurred and (b) the types of benefits they derived from their experiences	Mixed methods	Generic qualitative	Instrument with open- and closed-ended questions	Has anything positive come from you having had breast cancer?	Thematic analysis
Frye [49] (USA)	To learn how very long-term survivors identify in relation to cancer, how they have changed, both positively and negatively, and how they make meaning of their cancer experience	Qualitative	Phenomenology	Semi-structured interview	How have you changed as a result of your cancer experience? Please tell me in what ways you believe you are now different compared to how you were before you were diagnosed with cancer	Phenomenological analysis
Triplett [50] (USA)	To examine PTG and coping, QoL, depression, and social support in a sample of young breast cancer survivors	Mixed methods	Generic qualitative	Open-ended questionnaire	What positive experiences have you had?	Constant comparison
Connerty et al. [51] (Australia)	To explore people’s experiences of cancer to assess the relevance of the PTG construct and to identify potentially modifiable factors that may promote PTG	Qualitative	Generic qualitative	Semi-structured group interviews	Not reported	Thematic analysis
Fauske et al. [52] (Norway)	To explore how survivors of osteosarcoma of the lower extremity experience physical and psychosocial late effects several years after undergoing arduous treatment	Qualitative	Phenomenology	Semi-structured interview	Not reported	Thematic analysis
Mehrabi et al. [53] (Iran)	To investigate experiences relating to PTG of Iranian women with breast cancer	Qualitative	Phenomenology	Semi-structured interview	Not reported	Thematic analysis
Cheng et al. [54] (China)	To explore the perceptions of negative and positive life changes following treatment completion among breast cancer survivors	Mixed methods	Generic qualitative	Semi-structured interview	To what extent do you feel that your life has changed as a result of breast cancer and its treatment?	Content analysis
Martino et al. [55] (Italy)	To highlight the narrative markers and their transformative functions, underlying the PTG within cancer survivors’ narratives	Mixed methods	Narrative	Life story interview	Not reported	Other technique
Matheson et al. [31] (UK)	To examine younger testicular cancer survivors adjustment to survivorship	Qualitative	Grounded theory	Semi-structured interview	Not reported	Constant comparison
Hoogland [32] (USA)	To evaluate posttraumatic change in older adults with cancer by examining PTG and distress	Mixed methods	Generic qualitative	Open-ended questionnaire item	Not reported	Content analysis
Barthakur et al. [33] (India)	To understand the phenomenon of PTG in women survivors of breast cancer from an Indian perspective	Qualitative	Phenomenology	Semi-structured interview	Not reported	Phenomenological analysis
Raque-Bogdan et al. [34] (USA)	To examine how women diagnosed with breast cancer before the age of 40 made meaning of the impact of their cancer experience	Qualitative	Generic qualitative	Semi-structured interview	Not reported	Other technique
Adorno et al. [35] (USA)	To gain perspective on the scope of positive aspects perceived by a range of cancer survivors	Mixed methods	Grounded theory	Open-ended questionnaire item	Please tell us about any positive aspects of having cancer	Content analysis
Inan et al. [36] (Turkey)	To explore the nature of PTG in Turkish breast cancer survivors in the post-treatment first 2 years	Qualitative	Phenomenology	Semi-structured interview	Could you please tell me what positive life changes associated with breast cancer you have had?	Phenomenological analysis
Fallah et al. [37] (Iran)	To understand the dimensions of Iranian women’s perception of PTG among breast cancer patients	Qualitative	Phenomenology	Open-ended questionnaire	Not reported	Phenomenological analysis

#### Terms used to describe post-traumatic growth

Eighteen studies used the term post-traumatic growth; the remaining studies used a diverse range of terms (Online Resource Table 2). All definitions mentioned positive change, but the nature of any change was inconsistently defined; terms used included ‘psychological’, ‘meaningful’, or ‘mental’ change, ‘emotional growth’, and ‘changed sense of self’. Only one study (which used the term PTG [45]) mentioned the need for time to pass for PTG to develop. Three studies [35, 38, 45] overtly mentioned a ‘process’ or ‘journey’ of change within their definitions.

#### Study samples

Studies on breast cancer — and therefore, women — dominated (*n* = 14) (Table 1). Of the remaining studies, six included survivors of mixed cancer sites, four included survivors of haematological cancer, two were with head and neck cancer survivors, while there was one study of bone cancer and one of testicular cancer. Twelve studies were conducted in the USA, three in Australia, and two each in Iran and the UK; the remaining nine were each from a different country. Due to differences in study design and data collection methods, sample sizes ranged from six to 5149; 20 studies included ≤ 50 participants and 11 included ≤ 15. Studies varied in the time that had elapsed between cancer diagnosis or treatment and data collection. Within individual studies, between participant differences were common: for example, Cheng et al. [54] reported that time since diagnosis was anywhere between 6 months and 15 years. All studies were cross-sectional, collecting data at one point in time.

#### Study orientation and techniques

Sixteen studies were purely qualitative, while the other 12 used mixed methods. Fourteen purely qualitative studies conducted individual interviews, mostly described as semi-structured. One study carried out group interviews [51], while one described collecting open-ended written responses [37]. Of the mixed methods studies, seven conducted interviews as a separate qualitative component, while the other five used an open-ended questionnaire (two studies) or an open-ended item at the end of the quantitative component (e.g., text box at end of survey; three studies).

Thirteen studies did not report a methodological standpoint or philosophical position; these were labelled as taking a generic qualitative approach. Where a philosophical approach was named, the most common standpoint was phenomenology (nine) or grounded theory (three). Two studies took a narrative approach, and one a case study approach.

Sixteen studies (six purely qualitative; ten mixed methods) provided one or more examples of questions used to elicit qualitative data (Table 3). Seven studies reported asking quite broad questions relating to how life had changed following cancer [38, 41, 45–47, 49, 54]; six stated that they explicitly asked participants to discuss any positive changes they had experienced [35, 40, 43, 44, 48, 50]; example questions from remaining studies tended to refer to more specific changes, e.g., relationships with others [28, 54] or personal strength [29].

The most described analysis techniques were thematic analysis (nine studies), phenomenological analysis (four), content analysis (five), or constant comparison (four). Other approaches included framework (two), narrative analysis (one), and discourse/text analysis (one). Two studies, classified in Table 3 as ‘other’, used techniques described as redemptive sequence analysis of narrative markers [55] and a published coding process technique [34].

### Findings in relation to PTG outcomes

Online Resource Table 3 maps study findings to Tedeschi and Calhoun’s five PTG outcomes. Sixteen studies had findings within all five PTG outcomes; six studies mapped on to four outcomes; four studies mapped on to three outcomes, and two studies mapped onto two outcomes. There were no findings which did not map onto one of these outcomes.

#### Relating to others

Twenty-six studies reported findings relevant to the ‘Relating to others’ outcome. Survivors described how their experience had helped them prioritise, and improve, important relationships: ‘*It is one of the best things that has ever happened to me; it made family relationships better*’ [41, p6]. Survivors acknowledged that relationships with others are likely to change over the course of their cancer journey, and this helped them recognise who is important and who they should value*.*

Some reported a new willingness to express feelings and to understand complex emotions of others: ‘*Since developing the cancer, I can understand others’ emotions like pain. I work with disabled people now, I cheer them up in my heart, saying things like walk forward step by step in your life*’ [40, p111]. Development of empathy helped survivors connect with others in similar situations and to appreciate that everyone has their own challenges: ‘*I have become less judgemental, I think in a way that each person has their own story. You never know what people have experienced or gone through. If something happens to someone, I put myself in his or her situation. I think about how others experience things*’ [45, p6086].

#### New possibilities

Findings from 23 studies mapped on to the ‘New possibilities’ outcome. Some survivors reported they had taken a new life path, re-evaluating their career and choosing to pursue other possibilities: ‘*I had a complete lifestyle change now. I mean, I became (employment position) for my business and chucked it in and spent the last year doing support work*’ [30, p286]. Similarly, participants describe re-prioritising what is important in life, often deciding to spend (more) time with family and friends: ‘*Decided that it was more important to schedule my life to spend time with friends than to spend time at work*’ [53, p1417].

Health behaviour change was commonly reported as a positive change after cancer. Survivors described how they had learned to put themselves first and now chose to do things in their own best interests: ‘*I take better care of myself, [I am] more likely to say no to things*’ [41, p6]. Participants described lifestyle changes, ceasing unhealthy habits and adopting healthy eating and/or being more physically active. ‘*I thought if I continued the same lifestyle as before I got breast cancer, I would develop cancer again…I had been careless about foods…I have cut down the salt and limited my calorie intake and started avoiding some bad foods…*’ [40, p112]. A healthy lifestyle was sometimes perceived as a way to spend more time with family and friends: ‘*I have started an exercise program and am very healthy and want to stay in shape to do things with my daughter*’ [43, p64].

#### Personal strength

Twenty-seven studies reported findings within the ‘Personal strength’ outcome. Survivors reported strong feelings of self-efficacy following their cancer experience, and a belief that, if they can overcome cancer, then they can manage future challenges: ‘*Going through the experience with leukaemia, I grew even more confident, and we were talking about this and we think it is because I’d done the worst that life could dish out… I can do anything now*’ [30, p283]. Participants more resilience in day-to-day life: ‘*I can handle more. Can handle things better than others might do. Learned to speak up a little more and have become a little tougher*’ [45, p6086].

Some survivors described how their cancer journey had prompted a more positive attitude: ‘*Breast cancer made me a more positive person; a better person*’ [44, p339]. Self-acceptance grew, through identification as a cancer survivor and acknowledgement that what they had overcome was no small matter. This helped them adapt and/or rediscover, or find new, life pleasures: ‘*Being happy with myself, I accept what I am. I’ve changed my appearance. I discovered fashion, sexy underwear; sex again, my marriage, my husband, and everything. It was like being born again. I’m a success. I accept myself now… it’s freed me from that feeling guilty about things*’ [35, p672].

#### Appreciation of life

Findings relevant to the ‘Appreciation of life’ outcome were reported in 25 studies. Survivors described how cancer had given them a greater appreciation of good health and a second chance at life: ‘*I understand the value of life along with my spouse and children. Also I perceived health is valuable and we should appreciate it*’ [45, p1243]. Enhanced appreciation of the beauty in life and being (more) grateful for small things were frequently reported: ‘*A heightened sense of appreciation of people and things of beauty*’ [52, p1416].

For some, cancer survivorship brought into perspective the importance of living in the moment. They described appreciating the positives in life because they had already been through worse: ‘*Problems don’t worry me as much. I look at the beauty of life more than I used to before*’ [34, p674].

#### Spiritual change

Findings from 19 studies mapped to the ‘Spiritual change’ outcome. The predominant finding was that survivors’ spiritual beliefs strengthened and/or faith deepened. Some survivors’ beliefs led them to see having had cancer as something positive: ‘*My religious or spiritual beliefs… I feel much more confident they have been cemented a little bit more… I feel really strongly that I was really blessed*’ [36, p931]. Finding a deeper faith also helped cancer recovery: ‘*One thing that I did find as a positive after which was that my Christian faith deepened very significantly and I believe that was a very major factor in my recovery*’ [44, p339]. Survivors also credited their faith for their ability to manage challenges in day-to-day life: ‘*I have learned that my faith in God does make all things doable… good or bad*’ [43, p64]. Experiencing cancer also introduced some people to religion: ‘*It has led me to God. I learned a lot about myself, my life, and letting go of all those problems I was dealing with and looking at them in a different way*’ [41, p6].

## Discussion

By determining the nature and scope of the current qualitative evidence base on PTG in cancer, our intention was to identify any gaps in the current literature. As with research in other areas of cancer survivorship, studies on experiences following breast cancer or of mixed groups of cancer survivors dominate the literature. This means evidence is lacking on how PTG might differ across different cancer populations. Breast cancer, for example, is associated with higher socio-economic status and 5-year survival is high [56]. It has been suggested that PTG is influenced by the individual’s role pre-cancer and how their personal circumstances affect resilience and reaction to threat [14]. Socio-economic circumstances may, therefore, influence perception of trauma or ability to successfully mediate distress and hence impact potential for PTG. Cancer treatment experiences also vary by site (and stage); some treatments may alter appearance and lead to distress and social difficulties [57] while others can be invisible but still have long-term negative consequences (e.g., colostomy) [58]. Feelings of stigma and shame associated with some cancers (e.g., lung) may influence emotional and psychological reactions to the diagnosis [59, 60]. These issues, and the likely prognosis of the cancer, may all impact potential for, and experiences of, PTG. Research is warranted in more diverse patient populations — to explore all of this further.

Due to the dominance of breast cancer studies, the current literature mostly captures women’s experiences of PTG. Quantitative studies in cancer, and studies of other traumatic events, have suggested that women have higher levels of PTG than men [13, 61]. The reasons for this are not understood and might include differences between men and women in coping, appraisal of stressors, or cultural expectations; qualitative research in male cancers (e.g., prostate) or studies which seek to compare PTG experiences in men and women with the same cancer could shed light on these issues.

Cultural factors may influence PTG [62]. However, the studies identified here were mainly from highly developed Western countries (e.g., USA and Australia). Social networks, support, and integration will vary between countries [63]. The lack of cultural breadth in the current literature therefore limits potential to understand the influence of culture or other environmental factors on PTG.

Our scrutiny of study aims alongside methodological orientation indicates that the qualitative literature has largely focused on exploring and interpreting the lived experience of positive change following cancer. There was considerable variation in data collection methods, from unstructured interviews to open-ended questions placed at the end of surveys. Methodological details were commonly lacking. For example, many did not provide sample questions posed to interviewees, which could have provided insight into why findings were more focused on particular areas of PTG. Time since cancer diagnosis was inconsistently reported, despite being important for clarifying how and when PTG develops (e.g., how long do survivors struggle, reflect, or ruminate before PTG starts to develop?). Moreover, as all studies were cross-sectional (and most studies represented the combined experiences of survivors at different times from diagnosis), little is known about what initiates and sustains PTG, or how PTG manifests evolves over time or across the survivorship trajectory.

Our analysis of study findings demonstrates that PTG after cancer is seen across all five existing PTG outcomes, although not all outcomes emerged in every study. Many studies reported positive changes related to health behaviours. This is notable, given growing data on the role of lifestyle in cancer survival [64], and suggests that many may be receptive to advice and support around lifestyle change. However, such advice and support may need to be offered after someone has had time to reflect, rather than soon after diagnosis or end of treatment. Taking better care of one’s own health and physical wellbeing appeared to be intertwined with other positive changes, most notably greater appreciation of life. The role of rumination and reflection in facilitating health behaviour change post-cancer, and links between behaviour change and other positive changes, are worthy of further exploration both in and of themselves, and in relation to the design and implementation of behaviour change interventions.

Although we found that all positive changes reported in studies could map to the existing PTG outcomes, it is important not to infer that the model therefore captures the full extent of psychological changes that may occur. The experience of PTG following cancer is distinct from other traumatic experiences where the trauma is acute (e.g., accident or naturally disaster) and although the growth outcomes may be broadly similar, the processes and influences may differ. Moreover, as we note earlier, the current evidence base includes a narrow group of cancers and countries and cultures; widening the evidence base could uncover changes and experiences that fall out with the current framework.

## Limitations

To maintain a focus on PTG, we excluded studies that aimed to explore broader aspects of cancer survivorship experiences, and which might have reported, within other findings, one or more positive changes following cancer. These studies could, potentially, help fill some of the evidence gaps identified. We did not have resources for translation, and this may have contributed to the dominance of studies from Western countries.

## Conclusions

Qualitative research provides strong confirmation that PTG is experienced by cancer survivors and illuminates the lived experience of positive psychological change. However, there are important gaps in evidence. Future studies should prioritise populations that are currently underrepresented (e.g., cancers with poorer prognosis; cancers which occur more in lower socio-economic groups, diverse cultural experiences) and seek to better understand gendered experiences of PTG in cancer survivorship. Longitudinal research would be particularly valuable to explore timing and trajectories of PTG post-cancer, and what influences this.

## Supplementary Information

Below is the link to the electronic supplementary material.

Supplementary file1 (PDF 313 KB)

## Data Availability

Not applicable.
