# Supplementary material for: Post-traumatic growth after cancer: a scoping review of qualitative research
Source: Support Care Cancer. 2021 May 20;29(11):7013–27. doi: 10.1007/s00520-021-06253-2 (PMC8464569; doi:10.1007/s00520-021-06253-2)
Supplement: Supplementary file 1 — Supplementary file1 (PDF 313 KB) [file 520_2021_6253_MOESM1_ESM.pdf]

Post-traumatic growth after cancer: A scoping review of qualitative research.

Menger, Fiona. School of Education, Communication and Language Sciences, Newcastle University, UK (Corresponding author). fiona.menger@ncl.ac.uk

Mohammed Halim, Nurul Asyiqin, Newcastle University Medicine Malaysia, Tohor, Malaysia

Rimmer, Ben, Population Health Sciences Institute, Newcastle University Centre for Cancer, Newcastle University, UK

Sharp, Linda, Population Health Sciences Institute, Newcastle University Centre for Cancer Newcastle University, UK

Contents

Online Resource 1. Search strategy for MEDLINE. 2

Online Resource 2. Key terms used for PTG, definitions provided by study authors, and mapping of definitions on to Tedeschi and Calhoun's definition of PTG. 3

Online Resource 3. Qualitative findings mapped to Tedeschi and Calhoun's posttraumatic growth outcomes. 6

Online Resource 1. Search strategy for MEDLINE.

Key area for inclusion	Subject Headings	Keywords
Post-traumatic Growth	Adaptation, psychological/ or posttraumatic growth, psychological/	post-traumatic growth*, posttraumatic growth*, perceived benefit*, benefit-finding*, stress-related growth*, adversarial growth*, existential growth*, psychological growth*, self-transformation*, positive psychological change*, positive change*, thriv*, personal growth*, positive psychological outcome*
Cancer	Neoplasms/	neoplasm*, cancer*, tumo?r*
Participants who had sufficient time for rumination and reflection.	Exp Survivorship/, Exp Cancer Survivors/, Exp Aftercare/	Post treat*.ti,ab,kw, Survivor*.ti,ab,kw, (treat* adj complet*).ti,ab,kw, aftercare*.ti,ab,kw.
Qualitative Methods	Qualitative research/, Interview/ Focus group/mt, Grounded theory/, Personal narrative/, Autobiography/	interview*, focus group*, thematic analys#s, narrative*, mixed method*, content analys#s, grounded theor*, visual method*, interpretive phenomenological analys#s, autobiographical, ethnography, phenomenolog*

Online Resource 2. Key terms used for PTG, definitions provided by study authors, and mapping of definitions on to Tedeschi and Calhoun's definition of PTG.

Key Term[1] (number of papers)	*Citation number*	*Paper definition*	*Unplanned and unexpected*	*Emotional, behavioural, or cognitive change*	*Life crisis and struggle*	*Reflection and rumination*	*Growth is a process*	*Positive change*
Posttraumatic growth (18 papers)	31	How their interpersonal lives and perceptions have changed, both positively and negatively.		+				+
	32	Posttraumatic growth encompasses the positive changes that a person experiences following a traumatic event or upon overcoming a serious life crisis. Posttraumatic growth means that life becomes fuller, richer, and more meaningful.		+	+			+
	34	Positive psychological change as a result of a struggle with a traumatic event.		+	+			+
	37	Dynamic psychosocial process that occurs over time as the person is confronted with the multitude of changes elicited by the cancer and subsequent treatments.			+	+	+	
	38	The experience of individuals whose development, at least in some areas, has surpassed what was present before the struggle with crises occurred.			+			+
	39	Positive psychological changes experienced as a result of the struggle with highly challenging life circumstances.		+	+			+
	40	Positive changes following a cancer diagnosis.			+			+
	42	Positive psychological changes experienced as a result of the struggle with highly challenging life circumstances.		+	+			+
	43	Positive ways in which people have grown psychologically or emotionally from the cancer experience.		+	+			+
	44	Positive psychological change experienced as a result of the struggle with highly challenging life circumstances.		+	+			+

Key Term[1] (number of papers)	*Citation number*	*Paper definition*	*Unplanned and unexpected*	*Emotional, behavioural, or cognitive change*	*Life crisis and struggle*	*Reflection and rumination*	*Growth is a process*	*Positive change*
	45	A changed sense of oneself, a changed sense of one's relationship with others, and a changed philosophy of life.		+				
	46	Positive psychological change experienced as a result of the struggle with highly challenging life circumstances.		+	+			+
	48	The possibility that traumatic events may lead to a positive change/transformation.			+			+
	50	Any self-reported positive change resulting from coping with a traumatic event.			+			+
	51	In the face of challenges or traumas, individuals experience positive changes.			+			+
	52	The positive life changes that occur following a stressful or traumatic life event.			+			+
	54	Individuals' experiencing of meaningful positive changes arising from their struggles with major life difficulties.		+	+			+
	55	Positive mental changes that occur following traumatic events.		+	+			+
Benefit finding (3 papers)	33	People with cancer finding ways in which the cancer experience has enhanced their lives.			+			+
	35	The positive changes that result from the trauma of being diagnosed with a potentially life-threatening disease.			+			+
	53	A process such as meaning making or positive reappraisal that facilitates coping with distress or personal growth following some type of adversity.		+	+		+	+
Positive change(s) (2 papers)	30	Positive changes to their sense of personhood that resulted from having been forced to undertake the spiritual journey with a serious illness.		+	+		+	+

Key Term[1] (number of papers)	*Citation number*	*Paper definition*	*Unplanned and unexpected*	*Emotional, behavioural, or cognitive change*	*Life crisis and struggle*	*Reflection and rumination*	*Growth is a process*	*Positive change*
	36	Long-term positive life changes in survivorship of a serious illness.			+			+
Positive life change(s) (1 paper)	47	Experiences of positive changes to life following a cancer diagnosis.			+			+
Positive adjustment (1 paper)	49	Dismantling the threats of cancer to gain a sense of perspective and strive to restore normality.		+	+			
Perceived benefits (1 paper)	41	Anything positive that has come from them having breast cancer.			+			+
Self-transformation (1 paper)	29	Self-transformation encompasses both losses and growth (or other changes) in the self that may occur following diagnosis with an illness.			+			
Positive psychosocial sequelae (1 paper)	28	Positive psychosocial outcomes experienced following a life-threatening event.		+	+			+

[1]If a paper used multiple terms to describe posttraumatic growth, the most prominent term that was clearly defined in the paper, is the term included in the table.
'+' indicates that the definition given by the paper includes the component of the definition for posttraumatic growth, given by Tedeschi and Calhoun.

Online Resource 3. Qualitative findings mapped to Tedeschi and Calhoun's posttraumatic growth outcomes.

	**Posttraumatic growth outcomes**				
Source	*Relating to others*	*New possibilities*	*Personal strength*	*Spiritual change*	*Appreciation of life*
Fromm et al. [28]	+	+	+	+	+
Carpenter et al. [29]		+	+		
McGrath [30]	+	+	+	+	+
Dahan et al. [31]	+	+	+	+	+
Ruf et al. [32]	+	+	+		+
Helgeson [33]	+		+	+	+
Sadler-Gerhardt et al. [34]	+	+	+	+	+
Thambyrajah et al. [35]	+	+	+	+	+
Bishop et al. [36]	+	+	+	+	+
Morris et al. [37]	+	+	+		+
Hoggan [38]	+	+	+	+	+
Lelorain et al. [39]	+		+		+
Tsuchiya et al. [40]	+	+	+		+
Documet et al. [41]	+	+	+	+	+
Frye [42]	+	+	+	+	+
Triplett [43]	+	+	+	+	+
Connerty et al. [44]	+	+	+	+	+
Fauske et al. [45]	+	+	+		+
Mehrabi et al. [46]	+	+	+	+	+
Cheng et al. [47]	+	+	+		+
Martino et al. [48]	+	+	+	+	+
Matheson et al. [49]	+		+		
Hoogland [50]	+			+	+
Barthakur et al. [51]	+	+	+	+	+
Raque-Bogdan et al. [52]	+	+	+		
Adorno et al. [53]	+	+	+	+	+
Inan et al. [54]	+	+	+	+	+
Fallah et al. [55]			+	+	+

[a]'+' means that the outcome is either overtly reported by the paper or has been mapped to an existing outcome in our analysis.
